# A COMMUNITY HEALTH WORKER–LED HOME VISIT INTERVENTION TO SUPPORT DIVERSE DEMENTIA FAMILY CAREGIVERS

**DOI:** 10.1093/geroni/igac059.323

**Published:** 2022-12-20

**Authors:** Jung-Ah Lee, Eunae Ju, Eilleen Sabino-Laughlin, Lisa Gibbs, Adey Nyamathi

**Affiliations:** University of California, Irvine, Irvine, California, United States; University of California Irvine, Irvine, California, United States; UC Irvine, Irvine, California, United States; University of California, Irvine, Orange, California, United States; UC Irvine, Irvine, California, United States

## Abstract

Diverse family caregivers of persons with dementia (PWD) are often faced with challenges in obtaining necessary resources due to limited English-proficiency, accessing available resources, and stigma. Diverse community health workers (CHWs) provided dementia family caregivers culturally and linguistically appropriate education, compassionate listening during a 3-month home-visit intervention. 25 participants were recruited from communities in California: Race/Ethnicity 32% Korean, 32% Vietnamese, 16% Latino, 20% White; 76% Female; 44% Spouse; Age=62.6 (28-83 years); 44% work; English proficiency=2.9 of 5. Recruitment is ongoing. The key themes from the exit interview with participants included (a) accepting the role of a caregiver, (b) better understanding and patience with the PWD, and (c) ongoing support from CHW. The overall satisfaction on the intervention was 4.7/5. The CHW-led home-visit intervention was well-received by ethnically diverse dementia family caregivers. Participants reported the usefulness of education, community resources, and compassionate support delivered in their homes over 3 months.

